# Impaired inactive limb blood flow regulation in adults with multiple sclerosis during sympathoexcitatory stimuli

**DOI:** 10.14814/phy2.70694

**Published:** 2025-12-07

**Authors:** Sara R. Sherman, Natalia S. Lima, Robert W. Motl, Anthony T. Reder, Tracy Baynard, Bo Fernhall, Brooks A. Hibner

**Affiliations:** ^1^ Integrative Physiology Laboratory, Department of Kinesiology and Nutrition University of Illinois Chicago Chicago Illinois USA; ^2^ Department of Neurology‐Biological Sciences Division University of Chicago Chicago Illinois USA; ^3^ Department of Exercise and Health Sciences University of Massachusetts Boston Boston Massachusetts USA

**Keywords:** autonomic dysfunction, central regulation of blood flow, exercise, multiple sclerosis, peripheral regulation of blood flow, sympathoexcitatory stimulus

## Abstract

Individuals with multiple sclerosis (MS) exhibit reduced physical capacity, partly due to autonomic dysfunction. Autonomic impairments are well documented, but peripheral blood flow regulation during exercise remains unclear. We compared peripheral vascular responses to dynamic handgrip exercise, with and without a mild sympathoexcitatory stimulation, in individuals with and without MS. Twenty‐one individuals with MS (M/F: 4/17; 43 ± 10 yrs) and 27 individuals without MS (M/F: 10/17; 28 ± 6 yrs) completed a 5‐min supine dynamic handgrip exercise at 15% and 30% maximal voluntary contraction, which were repeated during −20 mmHg lower body negative pressure (LBNP). BP was assessed using finger photoplethysmography, and brachial artery blood flow via ultrasound. Individuals with MS had increased forearm vascular conductance to the inactive limb at baseline and in response to exercise compared to controls (Baseline to 30% MVC *with* LBNP: MS: +32% vs. Non‐MS: −12%; *p* for interaction *<*0.05). These changes occurred despite no differences between groups for forearm vascular conductance of the inactive limb (*p* for interaction = 0.523), and an attenuated increase in MAP in the group with MS during LBNP (*p* for interaction = 0.024). These findings suggest impaired vasomotor regulation in individuals with MS, particularly in response to combined mild sympathoexcitatory stimuli, compared to individuals without MS.

## 1 INTRODUCTION

Multiple sclerosis (MS) affects the central nervous system and is the most common disabling neurological disorder of young adults (Gilmour et al., 2018; Rolak, 2003). Individuals with MS display a range of symptoms such as fatigue, autonomic dysfunction, decreased mobility, and a greatly reduced physical work capacity (Flensner et al., 2013; Langeskov‐Christensen et al., 2015; Rudroff et al., 2016). Physical work capacity is a significant predictor of decline in physical function, morbidity and mortality, making it an important health indicator and potential target in treatment for individuals with MS (Heady et al., 1961; Langeskov‐Christensen et al., 2015; Myers et al., 2002). As such, understanding the nature of such limitations in work capacity in this population is critical for the development of optimal interventions to improve quality of life.

At rest, individuals with MS exhibit impaired blood pressure regulation (Racosta et al., 2015). This has been demonstrated by attenuated carotid baroreflex‐mediated increases in blood pressure to a hypotensive stimulus (i.e., neck pressure) (Huang et al., 2016), as well as reduced resting muscle sympathetic nerve activity, and catecholamine levels compared to those without MS (Keller et al., 2014). Reduced sympathetic activity limits the ability to vasoconstrict vessels in non‐active tissues, hindering the appropriate redistribution of blood flow, which is critical during exercise (Fadel, 2013; Holwerda et al., 2015; Joyner & Casey, 2015). However, blood flow control during exercise is not well understood in individuals with MS.

Peripheral regulation of blood flow during exercise requires a delicate balance between sympathetic vasoconstriction and local vasodilation to ensure adequate skeletal muscle blood flow during exercise while still maintaining blood pressure (Joyner & Casey, 2015; Mueller et al., 2017; Thomas & Segal, 2004). Evidence suggests that during rest individuals with MS have impaired resistance artery function compared to otherwise healthy individuals without MS, resulting in an impaired ability to vasodilate (Keményová et al., 2015; Ranadive et al., 2012); however, it is unknown whether this vasodilatory impairment is present during exercise. Lower body negative pressure (LBNP) is a mild sympathoexcitatory stimulus that can be added to an exercise stimulus to further assess peripheral blood flow regulation under heightened sympathetic stress (Goswami et al., 2019). Some clinical populations with impaired resistance artery function such as those with hypertension, can maintain muscle perfusion during exercise by increasing blood pressure to levels exceeding those of normotensive controls (Dipla et al., 2017). In contrast, individuals with MS may have a diminished ability to increase blood pressure and heart rate during exercise (Hansen et al., 2013; Ng et al., 2000; Pepin et al., 1996; Senaratne et al., 1984; Thomaides et al., 1993), potentially limiting their ability to adequately perfuse working muscle during exercise.

This study compared peripheral blood flow regulation during dynamic handgrip exercise with and without the mild sympathoexcitatory stimulus of −20 mmHg LBNP between individuals with and without MS. We hypothesized that individuals with MS would exhibit reduced blood flow to the working limb and increased blood flow to the nonworking limb during dynamic handgrip exercise when compared to those without MS. Furthermore, we expected these differences to be exacerbated when exercise was combined with mild LBNP, thereby challenging the ability to vasodilate.

## 2 METHODS

### 2.1 Participants

Volunteers aged 18–45 years, with a body mass index (BMI) ≤40 kg/m^2^, were recruited for this study through local MS support groups, Chicago‐based neurology clinics, and word‐of‐mouth. Neurological disability was assessed using the Kurtzke's Expanded Disability Status Scale (EDSS) by a trained and certified assessor (Neurostatus level C), wherein 0 is absence of disability and 10 is death in 0.5‐point increments (Goldman et al., 2010; Kurtzke, 1983; Rudick et al., 2010). Only individuals with relapsing–remitting MS and an EDSS score ≤ 6.0, with sufficient mobility to complete study protocols were enrolled (Beiki et al., 2019; Kurtzke, 1983). Relapsing–remitting is the most common MS subtype, accounting for approximately 85% of cases (Noseworthy et al., 2000; Van Le et al., 2019), thereby improving generalizability and reducing disease‐related heterogeneity. Disease diagnosis and exercise clearance were provided by the MS participant's neurologist, and all participants with MS were free from a neurologic episodic relapse for at least 30 days prior to the study. All participants were non‐smokers, non‐marijuana users, and free from significant cardiovascular, pulmonary, or metabolic disease. Written informed consent was provided by all participants in the study. This study was approved by the Institutional Review Board at the University of Illinois at Chicago (2018‐0788) and conformed to the guidelines set forth by the Declaration of Helsinki, with the exception of registration in a database.

### 2.2 Experimental design

Peripheral regulation of blood flow and central blood pressure were assessed in individuals with and without MS during dynamic handgrip exercise, which also included the mild sympathoexcitatory stimuli of LBNP, using an observational, prospective study design that is briefly described below (and detailed in Figure S1). All participants reported to the same temperature‐controlled (22–24°C) laboratory following a 24‐h abstinence from alcohol, caffeine, and exercise and at least 4 h postprandial. All measurements were conducted at a consistent time of day to minimize diurnal variation in hemodynamics and other physiological variables. Height, weight, and waist circumference were measured to the nearest tenth and used to calculate BMI. Women completed urine pregnancy tests prior to a Dual Energy X‐ray Absorptiometry (DEXA; GE, Lunar iDXA; Madison, Wisconsin) scan for whole body lean muscle and fat mass assessments. To isolate the limb composition for the inactive and active forearms, a region of interest was identified from the distal top of the radial styloid process to the proximal radial head captured on the DEXA scan. The cardiovascular hemodynamic response to peak aerobic exercise is reported from this cohort in a prior manuscript from our lab (Hibner et al., 2024).

### 2.3 Unilateral dynamic handgrip exercise protocol

Participants assumed a supine position in an LBNP chamber (VUV Analytics, Inc.; Cedar Park, Texas), with their lower body sealed tightly at the waist. Both upper limbs were extended perpendicularly (~80°) at heart level and rested on fitted cushioned tables designed to allow for simultaneous manipulation of both arms. A handgrip dynamometer (JLW Instruments; Chicago, Illinois) was placed in the participants' right hand for completion of the test of maximal voluntary contraction (MVC). Participants were instructed to perform three isometric contractions, precluding movement from their biceps brachii. The highest of the three contractions was used as their MVC to calculate relative workloads of 15% and 30% for the unilateral dynamic handgrip exercise protocol. The contracting arm and wrist were secured with a vac‐lok pillow (CQ Medical; Orange City, Iowa) to maintain a consistent position throughout the protocol and prevent the handgrip dynamometer from touching the table.

Following a period of rest and instrumentation, participants performed 5 min of dynamic handgrip at 15% and 30% of their calculated MVC with 5 min of rest between sets. A custom gauge was projected on the ceiling above the participant, which was programmed to provide visual feedback on their force production ensuring that the appropriate intensities were achieved. Dynamic handgrip cadence was set to 15 contractions per minute, using a 2‐s contraction and 2‐s relaxation rhythm. Subjects were assisted with keeping the correct cadence using a metronome. The LBNP chamber was then taken to −20 mmHg, to evoke a mild sympathoexcitatory stressor (Goswami et al., 2019). Following 5 min of acclimation to the mild sympathoexcitatory stressor of LBNP, two more sets of dynamic handgrip exercise were conducted, again at 15% and 30% of MVC during the −20 mmHg LBNP stimulus. These last two sets of dynamic handgrip exercise followed the same protocol as the first two sets, for a total of four handgrip conditions throughout the entire protocol: 2× dynamic handgrip exercise (i.e., 15% and 30% MVC) without LBNP and 2× (i.e., 15% and 30% MVC) dynamic handgrip exercise with LBNP. These two intensities (i.e., 15% and 30% MVC) dynamic handgrip exercise with LBNP have been shown not to evoke increased sympathetic nerve activity, despite reductions in muscle oxygenation in the exercising muscle (Hansen et al., 1996; Watanabe et al., 2007). Furthermore, both conditions (i.e., with and without LBNP) were preceded by a Baseline condition, which represents the last minute of a 5‐min rest in the supine position.

### 2.4 Brachial artery blood flow

Brachial artery diameter and velocity were recorded simultaneously from the active (right arm) and inactive (left arm) using two separate high‐frequency (5–13 MHz) linear array probes (Hitachi Aloka, Alpha 7; Tokyo, Japan) from each participant. Images were acquired from the distal third of the upper limb, above the antecubital fossa, while maintaining an insonation angle of 60° (Limberg et al., 2020). Image files were collected and analyzed offline using automated edge‐detection software (FMD Studio, Cardiovascular Suite, QUIPU; Pisa, Italy, was primarily used. However, Vascular Tools, Medical Imaging Applications; Coralville, Iowa was used in instances when the intima could not be removed from the image), by the same investigator. Brachial artery blood flow (mL/minute) was calculated: mean blood velocity (cm/second) × π × $\frac{\text{brachial artery diameter}\;{\left(\mathrm{cm}\right)}^{2}}{4}$ × 60. Brachial artery vascular conductance (mL/minute/100 mmHg) was calculated: $\frac{\text{brachial artery blood flow}\;\left(\mathrm{mL}/\text{minute}\right)}{\text{mean arterial pressure}\;\left(\text{mmHg}\right)}\times 100$. Brachial artery vascular resistance (mmHg/mL/minute) was calculated: $\frac{\text{mean arterial pressure}\;\left(\text{mmHg}\right)\;}{\text{brachial artery blood flow}\;\left(\mathrm{mL}/\text{minute}\right)}$. Both active and inactive limb blood flow and conductance were normalized to their respective forearm lean muscle mass, assessed during the DEXA scan, but raw values are presented unless otherwise noted. In our laboratory, the coefficient of variation for brachial artery blood flow is ≤5% for both intra‐ and inter‐day measurements, and analysis checks during data capture/analysis confirmed that baseline blood flow and conductance remained within these expected limits.

### 2.5 Hemodynamic variables

Beat‐to‐beat heart rate (HR) and blood pressure (BP) were continuously recorded using a data acquisition system (Biopac; Santa Barbara, California) at 1000 Hz throughout the experimental protocol. Heart rate was recorded using a three‐lead electrocardiogram (ECG; BioPac, Santa Barbara, California). Beat‐to‐beat systolic BP (SBP), diastolic BP (DBP), pulse pressure (PP), mean arterial pressure (MAP), and stroke volume (SV) were collected using noninvasive finger photoplethysmography (Finometer Pro; TNO Biomedical Instrumentation; Enschede, Netherlands) on the inactive limb. The Finapres was recalibrated against brachial cuff pressures between handgrip sessions at baseline and –20 mmHg LBNP. Hemodynamic values were recorded after an adaptation period and stabilization at each condition, in addition to the last minute of the condition, as described in the unilateral dynamic handgrip exercise protocol ensuring that all variables were time aligned on the data acquisition system. Stroke volume was calculated via Modelflow (Harms et al., 1999) and adjusted to aortic diameter, which was measured at the left ventricular outflow track using a parasternal long‐axis view during systole with a phased array (2.5–5.2 MHz) cardiac ultrasound probe. Cardiac output (CO) is calculated as the product of HR × SV. Both SV and CO were indexed to body surface area using the DuBois formula (DuBois, 1916), and labeled SVindex and COindex, respectively. Total peripheral resistance (TPR) was calculated as: $\frac{\text{mean arterial pressure}\;\left(\text{mmHg}\right)\;}{\mathrm{CO}\;\left(L/\text{minute}\right)}\times 100$. All data files were downloaded and analyzed offline using separate data analysis software (PowerLab; ADI instruments; Colorado Springs, Colorado) by the same investigator.

### 2.6 Statistical analyses

Data are presented as (1) Baseline: the last minute of rest preceding the 15% MVC dynamic handgrip exercise condition; (2) 15% MVC: the last minute of dynamic handgrip exercise at 15% of MVC; (3) 30% MVC: the last minute of dynamic handgrip exercise at 30% of MVC. A separate analysis is conducted on the same three conditions for the −20 mmHg LBNP stimuli (i.e., Baseline with LBNP, 15% MVC with LBNP, and 30% MVC with LBNP). All main outcome variables (separately with and without LBNP) were assessed with a 2 (MS vs. Non‐MS) by 3 (Baseline, 15%MVC, 30%MVC) repeated measures analysis of variance to determine differences between groups given the different conditions of handgrip exercise with a Bonferroni correction for multiple comparisons. Due to the complexity of the study design, and the study question, we did not complete an analysis to compare both the active and the inactive limb. All data are presented as mean ± standard deviation and normality was assessed quantitatively using a Shapiro–Wilk test. Descriptive characteristics were compared using a one‐way analysis of variance (ANOVA) for continuous variables and χ^2^ tests for categorical data. Greenhouse–Geisser correction was used in the case where sphericity was violated. Supine baseline hemodynamic (i.e., blood flow variables such as brachial diameter and velocity descriptors) were analyzed using a *t*‐test for both the active and inactive limbs, in two separate group analyses. Analyses were performed using SPSS (version 28; IBM Corp; Armonk, New York) with *α* ≤ 0.05.

Although no data currently exist on skeletal muscle blood flow in individuals with MS, prior work from our lab (Ranadive et al., 2012) has described differences in peripheral arterial vasodilatory ability between individuals with and without MS (Cohen's *d* effect sizes for resting forearm blood flow: *d* = −0.61 and peak forearm blood flow following reactive hyperemia: *d =* −0.64). Utilizing a Type 1 error of *α* = 0.05 and power of 0.80, for a repeated‐measures ANOVA (*F*‐test), within‐between interaction, those data were used to conduct an a priori power analysis (G*Power; Heinrich Heine University, Düsseldorf, Germany) revealing a required sample of *n =* 17 per group for a large effect (*d* ≥ 0.80). Supplemental participants were added to minimize the chances of Type II error and presumed data loss. A secondary analysis of covariance was performed on all variables covarying for age, MVC and limb lean mass.

## 3 RESULTS

Twenty‐one individuals with MS and 26 individuals without MS completed this study and their descriptive characteristics are listed in Table 1. Individuals with MS were older than the individuals without MS (*p <* 0.001) and the median EDSS score for the individuals with MS was 3.5 (2). The majority (~90%) of participants from both groups were right‐hand dominant; however, limb dominance has not been shown to affect forearm hemodynamics during rhythmic handgrip exercise (Schwartz et al., 2024).

**TABLE 1:** Descriptive characteristics of the 48 participants who completed the study.

Variable	Non‐MS	MS	*p*
*n* (M/F)	27 (10/17)	21 (4/17)	0.214
Age (years)^a^	28 ± 6	43 ± 10	<0.001
Race/Ethnicity^a^	White: 7 Hispanic: 12 Latino: 3 Black: 1 Asian: 4	White: 3 Hispanic: 1 Latino: 14 Black: 2 Asian: 1	<0.001
Height (cm)	171.1 ± 8.5	168.3 ± 8.0	0.254
Weight (kg)	73.8 ± 15.9	73.6 ± 17.8	0.971
BMI (kg/m^2^)	25.0 ± 3.8	25.9 ± 5.8	0.495
Waist circumference (cm)	77.5 ± 11.0	83.5 ± 17.9	0.158
Hip circumference (cm)	98.7 ± 10.0	103.9 ± 13.6	0.134
Body fat (%)	30.9 ± 10.5	33.7 ± 10.2	0.356
Lean mass right arm (g, singular)	2931 ± 1066	2434 ± 739	0.075
MVC (kg)	79.2 ± 22.5	70.6 ± 20.5	0.177
15% MVC	11.9 ± 3.4	10.6 ± 3.1	0.177
30% MVC	23.8 ± 6.7	21.2 ± 6.1	0.177

*Note*: Data presented mean ± standard deviation.

Abbreviations: BMI, body mass index; F, female; M, male; MS, multiple sclerosis; MVC, maximal voluntary contraction.

^a^ Groups different.

### 3.1 Brachial artery blood flow

Baseline resting hemodynamic variables from both the active and inactive limbs are included in Table 2.

**TABLE 2:** Supine baseline resting hemodynamic variables.

Variable	*n*	Non‐MS	*n*	MS	*p*
Inactive arm					
Brachial diameter (mm)	26	3.83 ± 0.56	21	3.61 ± 0.71	0.245
Total velocity^a^ (cm/sec)^b^	26	10.2 ± 3.6	21	14.7 ± 5.0	<0.001
Antegrade velocity (cm/sec)^b^	26	12.1 ± 4.0	21	16.0 ± 4.9	0.004
Retrograde velocity (cm/sec)	26	−2.0 ± 2.4	21	−1.3 ± 1.8	0.316
Baseline blood flow (mL/min)^b^	26	66 ± 10	21	84 ± 15	<0.001
Active arm					
Brachial diameter (mm)	25	3.83 ± 0.53	20	3.71 ± 0.75	0.523
Total velocity^a^ (cm/sec)^b^	25	10.8 ± 3.2	20	15.4 ± 5.3	<0.001
Antegrade velocity (cm/sec)	25	15.5 ± 4.9	20	18.2 ± 5.2	0.077
Retrograde velocity (cm/sec)	25	−4.6 ± 3.4	20	−2.8 ± 3.0	0.055
Baseline blood flow (mL/min)	25	76 ± 13	20	82 ± 20	0.225

*Note*: Data presented mean ± standard deviation.

Abbreviations: MS, multiple sclerosis.

^a^ Total velocity = antegrade – retrograde.

^b^ Groups different.

#### 3.1.1 Active limb

**FIGURE 1:**
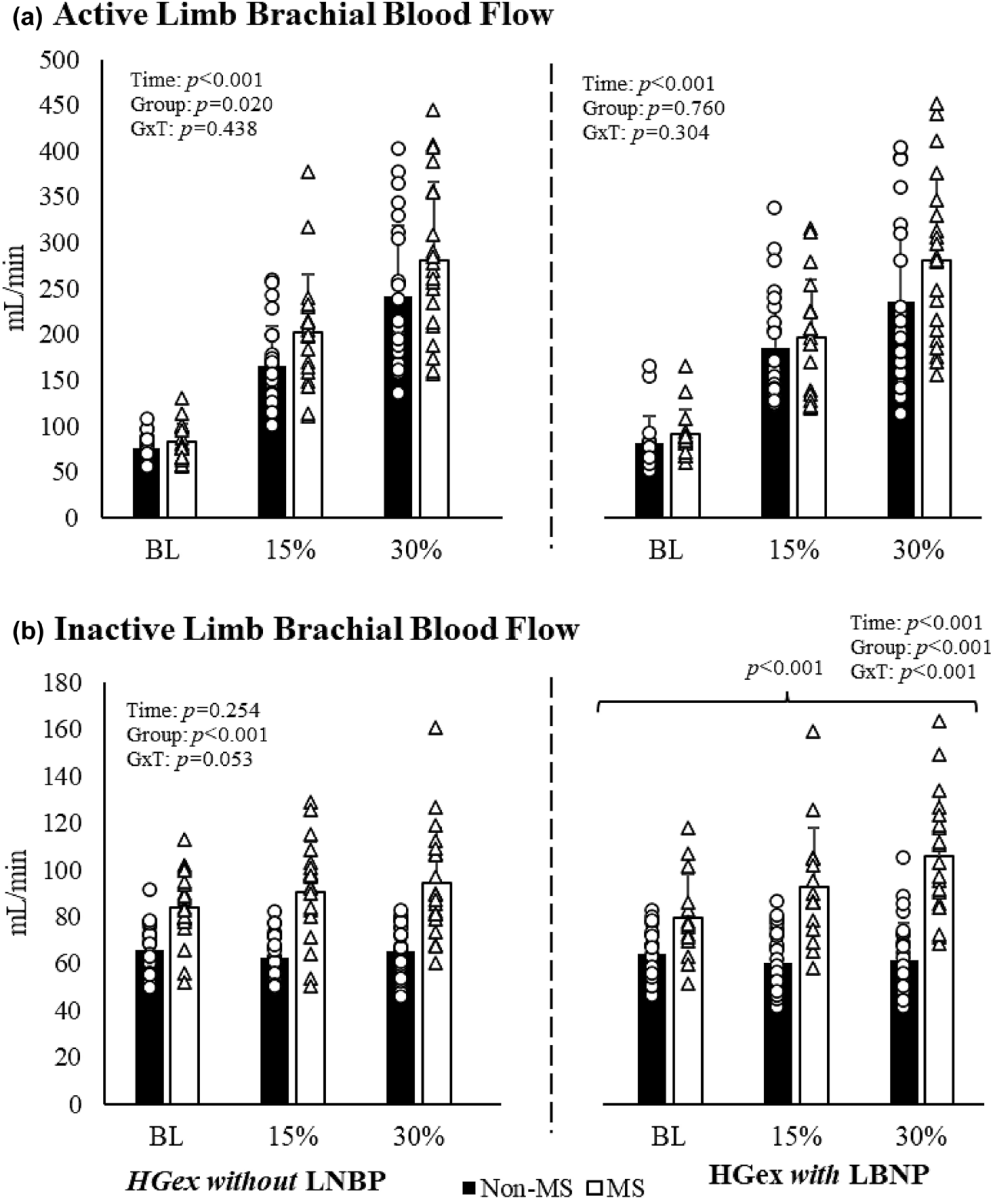
Active (a) and inactive (b) limb brachial blood flow variables across all timepoints where dark bars represent individuals without MS and open bars represent individuals with MS. The percentages below the bars represent the handgrip exercise intensity corresponding to baseline, 15%, and 30% of maximal voluntary contractions with (left of the dotted line) and without (right of the dotted line) the −20 mmHg LBNP stimulus. Individuals with MS have greater brachial blood flow in their inactive limb (b) at all three conditions of HGex with (right) LBNP compared to individuals without MS in a Bonferroni test for multiple comparisons. BL, baseline condition; G×T, group × time interaction; HGex, dynamic handgrip exercise; LBNP, lower body negative pressure; MS, multiple sclerosis; “15%” and “30%” represent relative intensities of maximal voluntary contraction; Data presented mean ± standard deviation.

Two individuals were excluded from active limb blood flow analysis in both groups due to the inability to collect adequate data. Thus, a total of 24 individuals without MS and 19 individuals with MS are included in the analysis for this variable. The individuals with MS had a greater overall brachial blood flow compared to individuals without MS across all handgrip exercise conditions without LBNP (i.e., Baseline, 15%, 30% MVC; *p* for group effect = 0.020; Figure 1a). However, this group effect was removed when covarying for age (*p* for group effect = 0.310), but not when considering MVC and active limb lean mass. Brachial artery blood flow was not different between the two groups during any of the handgrip exercise conditions with LBNP (Baseline with LBNP, 15% MVC with LBNP, and 30% MVC with LBNP; *p* for group × time interaction > 0.05).

#### 3.1.2 Inactive limb

Brachial artery blood flow to the inactive limb did not change across any of the handgrip exercise conditions without LBNP for either group (*p* for time effect> 0.05; Figure 1b). However, individuals with MS exhibited higher blood flow to the active limb and increased blood flow to the inactive limb during all three handgrip exercise conditions without LBNP compared to those without MS, but the differential response did not quite achieve statistical significance (*p* for group × time interaction = 0.053). Individuals with MS had greater blood flow to their inactive limb at all three conditions of dynamic handgrip with the −20 mmHg LBNP stimulus (*p* for group effect < 0.001). Furthermore, while the individuals without MS slightly decreased their blood flow to the inactive limb during the incremental bouts of handgrip with the LBNP stimulus, the individuals with MS slightly increased (~35% increase during 30% MVC with −20 mmHg LBNP) their blood flow to the inactive limb during the exercise bouts (with LBNP; *p* for group × time interaction < 0.001). These effects remained upon covarying for age, MVC, and inactive limb lean mass. Percentage change values for brachial artery blood flow in the active and inactive limbs are presented in Figure S2. Notably, at 30% maximal voluntary contraction, the inactive limb exhibited significantly greater percentage changes in individuals with MS compared with controls, both with and without LBNP.

### 3.2 Brachial artery vascular conductance

#### 3.2.1 Active limb

**FIGURE 2:**
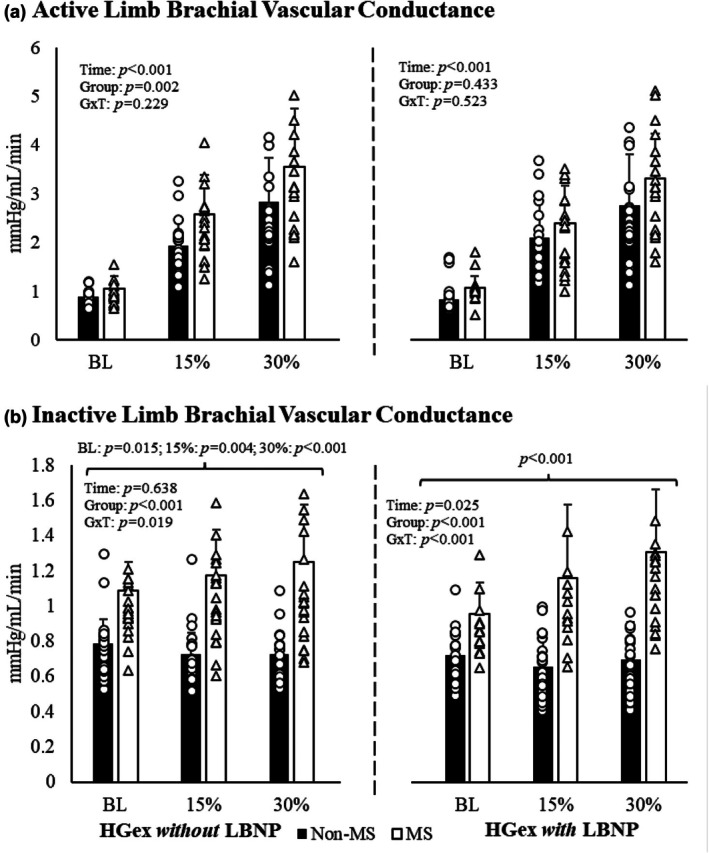
Active (a) and inactive (b) limb brachial vascular conductance across all timepoints where, as in Figure 1, dark bars represent individuals without MS and open bars are individuals with MS. The percentages below the bars represent the handgrip exercise intensity corresponding to baseline, 15%, and 30% of maximal voluntary contractions with (left of the dotted line) and without (right of the dotted line) the −20 mmHg LBNP stimulus. Individuals with MS have greater brachial blood flow in their inactive limb (B) at all conditions of HGex without (left) and with (right) LBNP compared to individuals without MS in a Bonferroni test for multiple comparisons. BL, baseline condition; G×T, group × time interaction; HGex, dynamic handgrip exercise; LBNP, lower body negative pressure; MS, multiple sclerosis; “15%” and “30%” represent relative intensities of maximal voluntary contraction; Data presented mean ± standard deviation.

Similar to the blood flow data, the individuals with MS had a greater overall brachial artery vascular conductance compared to individuals without MS for the exercise without LBNP conditions (*p* for time effect < 0.001; *p* for group effect = 0.002; Figure 2a). Both groups increased vascular conductance with handgrip exercise without LBNP with no significant interaction (*p* for group × time interaction = 0.229) for the term. However, the significant group effects were not present in the handgrip conditions with LBNP (*p* for group effect = 0.433; *p* for group × time interaction = 0.229). These results remained when covarying for age, MVC, and active limb lean mass.

#### 3.2.2 Inactive limb

Inactive‐limb vascular conductance did not change in individuals without MS and was overall lower in this group during handgrip exercise without LBNP compared to those with MS (*p* for group × time interaction = 0.019; Figure 2b). A similar but more pronounced pattern was observed under LBNP conditions. Specifically, while vascular conductance in the inactive limb remained unchanged in non‐MS participants, individuals with MS exhibited increased conductance across all conditions (baseline, 15%, and 30% MVC) during handgrip exercise with −20 mmHg LBNP (*p* for group × time interaction < 0.001). This effect persisted after adjusting for age, MVC, and inactive‐limb lean mass.

### 3.3 Brachial artery vascular resistance

#### 3.3.1 Active limb

**FIGURE 3:**
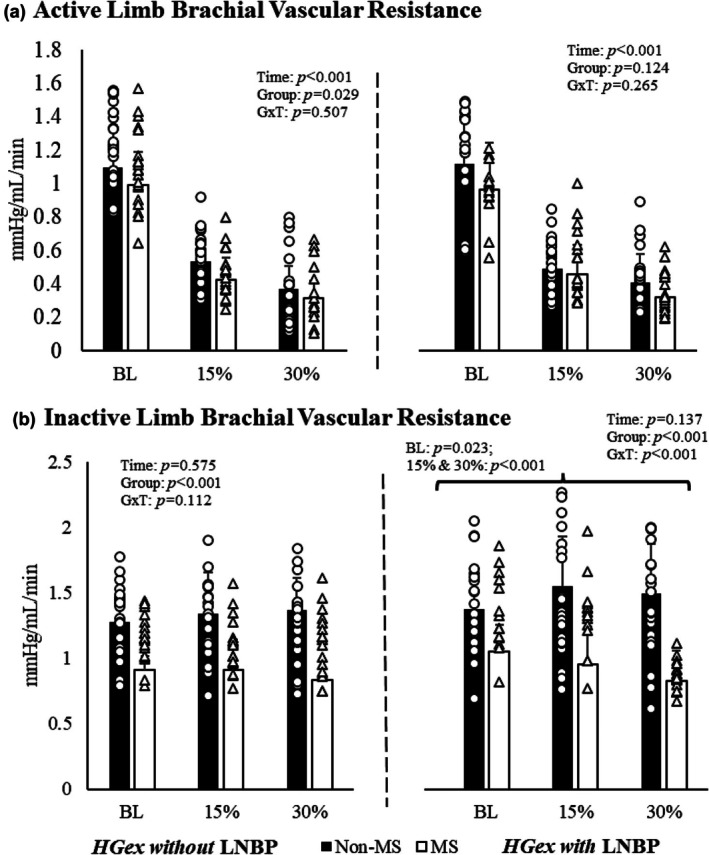
Active and inactive limb brachial vascular resistance across all timepoints where, as in Figures 1 and 2, dark bars represent individuals without MS and open bars are individuals with MS. The percentages below the bars represent the handgrip exercise intensity corresponding to baseline, 15%, and 30% of maximal voluntary contractions with (left of the dotted line) and without (right of the dotted line) the −20 mmHg LBNP stimulus. Individuals with MS have reduced brachial vascular resistance in their inactive limb (B) at all three conditions of HGex with (right) LBNP compared to individuals without MS in a Bonferroni test for multiple comparisons. BL, baseline condition; G×T, group × time interaction; HGex, dynamic handgrip exercise; LBNP, lower body negative pressure; MS, multiple sclerosis; “15%” and “30%” represent relative intensities of maximal voluntary contraction; Data presented mean ± standard deviation.

The individuals with and without MS had different brachial vascular resistance responses during handgrip exercise (*p* for group effect = 0.029) without LBNP (*p* for group × time interaction = 0.507; Figure 3a). However, there were no differences in vascular resistance during any of the three conditions for either group during handgrip exercise with LBNP (*p* for group × time interaction = 0.265). All of these differences remained when covarying for age, MVC, and inactive limb lean mass.

#### 3.3.2 Inactive limb

Similar to the active limb brachial vascular resistance results, the individuals with and without MS had different vascular responses in the inactive limb during handgrip exercise without LBNP, with no significant interaction for the term (*p* for group effect < 0.001; *p* for group × time interaction = 0.112; Figure 3b). However, the differences in brachial vascular resistance became more pronounced in the inactive limb during handgrip exercise with LBNP such that the individuals without MS had greater resistance throughout all three conditions (i.e., Baseline, 15% and 30% MVC) compared to the individuals with MS (*p* for group effect < 0.001; *p* for group × time interaction < 0.001). These effects remained when covarying for age, MVC, and inactive limb lean mass.

### 3.4 Hemodynamic variables

Two participants with MS were excluded from analysis during handgrip exercise without LBNP (Non‐MS: *n =* 27; MS: *n =* 19), and two additional participants from each group were excluded during handgrip exercise conditions with −20 mmHg LBNP (Non‐MS: *n =* 25; MS: *n =* 17) due to technical issues preventing adequate data collection. Mean arterial pressure was different overall between groups and timepoints, but both groups demonstrated a similar pattern of change over time for all three baseline conditions without −20 mmHg LBNP (*p* for time effect < 0.001; *p* for group effect = 0.011; Table 3). Furthermore, significant time and group effects were removed when covarying for age (*p* for time with covarying for age = 0.351; *p* for group with covarying for age = 0.344) in these three baseline conditions (baseline, 15% MVC, 30% MVC) without −20 mmHg LBNP; however, no differences were observed when covarying for BMI, and active limb lean mass between the groups higher at baseline in the individuals without MS compared to those with MS with the −20 mmHg LBNP stimuli (*p =* 0.023 in a Bonferroni test of multiple comparisons; *p* for group × time interaction = 0.024; Table 3). However, while MAP did not change for the individuals with MS during the handgrip exercise with −20 mmHg LBNP stimuli, the individuals without MS increased their MAP during the handgrip exercise at 30% of MVC (*p =* 0.021 in a Bonferroni test of multiple comparisons). Other blood pressure variables (SBP, DBP, and PP) were not different between groups during the handgrip exercise without LBNP conditions (*p* for group effect > 0.05; Table 3), with expected stable blood pressure variables across handgrip exercise without LBNP in both groups (*p* for group × time interaction > 0.05) (Hansen et al., 1996; Watanabe et al., 2007). Pulse pressure did not change during handgrip exercise without LBNP (*p* for time effect = 0.866). During the handgrip exercise with −20 mmHg LBNP stimuli SBP, DBP, and PP were not different between individuals with and without MS (*p* for group × time interaction > 0.05). These results remained when covarying for age, MVC, and active limb lean mass.

**TABLE 3:** Blood pressure variables for individuals with and without MS across all timepoints of handgrip exercise *with* (left) and *without* (right) the −20 mmHg LBNP stimulus.

	HGex without LBNP							HGex with −20 mmHg LBNP						
	*n*	Baseline	15% MVC	30% MVC	Time	Group	G × T	*n*	Baseline^a^	15% MVC^a^	30% MVC^a^	Time	Group	G × T
(A) Mean arterial pressure (mmHg)														
Non‐MS	27	84 ± 9	85 ± 9	89 ± 14	<0.001	0.011	0.742	25	86 ± 9	88 ± 9	90 ± 14	0.002	0.036	0.024
MS	19	78 ± 8	78 ± 7	82 ± 9				17	80 ± 6	82 ± 6	83 ± 6			

	*n*	Baseline	15% MVC	30% MVC	Time	Group	G × T	*n*	Baseline	15% MVC	30% MVC	Time	Group	G × T
(B) Systolic blood pressure (mmHg)														
Non‐MS	27	107 ± 19	108 ± 20	111 ± 21	0.099	0.159	0.670	25	109 ± 20	111 ± 20	111 ± 22	0.448	0.431	0.269
MS	19	99 ± 15	99 ± 14	104 ± 17				17	104 ± 15	104 ± 11	107 ± 6			

	*n*	Baseline	15% MVC	30% MVC	Time	Group	G × T	*n*	Baseline	15% MVC	30% MVC	Time	Group	G × T
(C) Diastolic blood pressure (mmHg)														
Non‐MS	27	70 ± 13	70 ± 13	72 ± 18	0.157	0.512	0.681	25	72 ± 11	74 ± 12	77 ± 14	<0.001	0.475	0.304
MS	19	67 ± 9	68 ± 8	70 ± 7				17	70 ± 11	72 ± 9	74 ± 6			

	*n*	Baseline	15% MVC	30% MVC	Time	Group	G × T	*n*	Baseline	15% MVC	30% MVC	Time	Group	G × T
(D) Pulse pressure (mmHg)														
Non‐MS	27	57 ± 12	57 ± 11	59 ± 11	0.866	0.564	0.229	25	56 ± 13	56 ± 12	57 ± 12	0.536	0.332	0.648
MS	19	61 ± 13	61 ± 14	58 ± 25				17	60 ± 14	60 ± 13	60 ± 13			

*Note*: Data presented as mean ± standard deviation.

Abbreviations: G×T, group × time interaction; HGex, dynamic handgrip exercise; LBNP, lower body negative pressure; MS, multiple sclerosis; MVC, maximal voluntary contraction.

^a^ Non‐MS higher than MS at time point, *p <* 0.05.

The individuals without MS increased COindex during the handgrip exercise without LBNP condition, while COindex did not change in the individuals with MS during this condition (*p* for group × time interaction = 0.003; Table 4b). Individuals with MS had higher COindex at Baseline and 15% MVC, but COindex was similar between groups at 30% MVC during handgrip exercise without LBNP. Cardiac output indexed to body surface area was not different between groups during the exercise with −20 mmHg LBNP conditions (Baseline with LBNP, 15% MVC with LBNP, and 30% MVC with LBNP; *p* for group effect = 0.214), and COindex increased similarly in both groups with handgrip exercise during the LBNP conditions (*p* for group × time interaction = 0.077). Stroke volume indexed to body surface area was not different between groups at any condition (Table 4c). These results remained when covarying for age, MVC, and active limb lean mass.

**TABLE 4:** Hemodynamic variables for individuals with and without MS across all timepoints of handgrip exercise *with* (left) and *without* (right) the −20 mmHg LBNP stimulus.

	HGex without LBNP							HGex with −20 mmHg LBNP						
	*n*	Baseline	15% MVC	30% MVC	Time	Group	G × T	*n*	Baseline	15% MVC	30% MVC	Time	Group	G × T
(A) Total peripheral resistance (100 mmHg/L/min)														
Non‐MS	27	13.7 ± 4.9	13.7 ± 5.0	14.3 ± 6.0	0.387	0.296	0.419	25	14.9 ± 6.0	16.3 ± 7.2	16.0 ± 7.3	0.015	0.828	0.506
MS	19	12.6 ± 3.2	12.3 ± 3.2	12.5 ± 3.9				17	13.8 ± 4.7	16.4 ± 7.0	15.6 ± 6.0			

	*n*	Baseline^a^	15% MVC^a^	30% MVC	Time	Group	G × T	*n*	Baseline	15% MVC	30% MVC	Time	Group	G × T
(B) Cardiac output index (L/m^2^)														
Non‐MS	27	3.3 ± 0.7	3.4 ± 0.7	3.7 ± 0.7	0.007	0.259	0.003	25	3.1 ± 0.7	3.2 ± 0.7	3.4 ± 0.8	<0.001	0.214	0.077
MS	18	3.9 ± 1.6	3.9 ± 1.5	3.9 ± 1.7				17	3.4 ± 1.5	3.5 ± 1.5	4.0 ± 1.5			

	*n*	Baseline	15% MVC	30% MVC	Time	Group	G × T	*n*	Baseline	15% MVC	30% MVC	Time	Group	G × T
(C) Stroke volume index (mL/m^2^)														
Non‐MS	27	55.0 ± 11.2	56.4 ± 11.6	58.2 ± 12.7	0.340	0.492	0.024	25	51.8 ± 12.3	52.5 ± 13.1	52.2 ± 13.6	0.370	0.606	0.620
MS	18	60.2 ± 19.3	60.5 ± 18.6	58.9 ± 20.9				17	54.1 ± 19.3	54.5 ± 17.8	55.4 ± 18.5			

	*n*	Baseline	15% MVC	30% MVC	Time	Group	G × T	*n*	Baseline	15% MVC	30% MVC	Time	Group	G × T
(D) Heart rate (bpm)														
Non‐MS	27	61 ± 7	62 ± 8	65 ± 8	<0.001	0.065	0.111	25	61 ± 8	62 ± 8	67 ± 9	0.006	0.010	0.197
MS	19	67 ± 13	68 ± 12	69 ± 13				17	71 ± 15	70 ± 10	73 ± 9			

*Note*: Data presented as mean ± standard deviation.

Abbreviations: bpm, beats per minute; G×T, group × time interaction; HGex, dynamic handgrip exercise; LBNP, lower body negative pressure; MS, multiple sclerosis; MVC, maximal voluntary contraction.

^a^ MS higher than Non‐MS at time point, *p <* 0.05.

Heart rate was not different between groups in the handgrip exercise without LBNP conditions and increased similarly during handgrip exercise in both groups (Table 4d). Heart rate was higher at Baseline in the individuals with MS during the handgrip exercise with LBNP conditions but increased similarly during exercise for both groups (*p* for group effect = 0.010; *p* for group × time = 0.197). Total peripheral resistance was not different between groups during the handgrip exercise without LBNP conditions and did not change during handgrip exercise in either group (Table 4a). However, while both groups steadily decreased TPR during exercise in the handgrip exercise with LBNP conditions, this decrease was significantly greater in the group with MS (*p* for time effect = 0.004; *p* for group × time interaction = 0.037).

## 4 DISCUSSION

We evaluated and compared peripheral muscle blood flow regulation during handgrip exercise with and without the influence of mild sympathoexcitatory stimuli (i.e., LBNP) between individuals with and without MS. Our main findings demonstrate that brachial artery blood flow in the exercising limb was higher in individuals with MS, both at rest and during handgrip exercise under standard conditions, without LBNP. These group differences disappeared when performing handgrip exercise with the −20 mmHg LBNP stimulus. However, the observed increase in blood flow was similar between individuals with and without MS. Conversely, blood flow in the non‐exercising limb (i.e., inactive arm), increased during exercise with the opposite limb in individuals with MS. However, this reciprocal increase did not occur in individuals without MS, and the observed increase in inactive brachial artery blood flow was exaggerated during handgrip exercise with the −20 mmHg LBNP stimulus. Our findings indicate that individuals with MS increase blood flow to the exercising limb during handgrip conditions appropriately, but lack the ability to appropriately control blood flow in inactive muscle during the single limb handgrip exercise with the −20 mmHg LBNP stimulus. While our data suggest that blood flow to exercising muscle may not be a limiting factor for exercise in individuals with MS, the lack of blood flow control in the inactive limb suggests that individuals with MS may not be able to appropriately redistribute blood flow to exercising limbs during exercise. This is unlikely to be a limitation during small muscle mass submaximal exercise, such as unilateral dynamic handgrip exercise, but could potentially be a greater issue during large muscle mass, high‐intensity exercise.

Our findings suggest that individuals with MS responded less appropriately to mild sympathoexcitatory stimuli, compared to those without MS. This aligns with previous studies reporting reduced spontaneous sympathetic nerve activity and lower resting plasma norepinephrine levels in individuals with relapsing–remitting MS, compared to age‐ and sex‐matched healthy individuals without MS (Ogoh et al., 2002). Additionally, individuals with MS have reduced carotid baroreflex responsiveness to hypotension (e.g., neck pressure), which appears to stem from a diminished ability to decrease vascular conductance (Huang et al., 2016).

The unilateral dynamic handgrip model used in the current study promotes an exercise‐induced hyperemic response that is not dependent on the ability to substantially increase CO (Joyner & Casey, 2015). Moreover, the inverse relationship between peripheral resistance and blood flow suggests the lack of vasoconstriction in the inactive limb, and the reduced blood pressure response to handgrip exercise during the −20 mmHg LBNP conditions in individuals with MS, potentially reflect changes in downstream vascular resistance. Compensatory blood pressure responses to carotid baroreceptor perturbation, as described above (Huang et al., 2016), are predominately driven by changes in peripheral vascular tone. The reduced blood pressure response to hypotensive perturbations suggests that there are impairments in vasomotor adjustments in MS. These impairments are potentially due to abnormal sympathetic modulation of blood vessels, a pattern that is observed in the current study (Kissell et al., 2024). While blood pressure variability is an indirect and limited indicator of sympathetic activity that was not captured in the current study, impaired sympathetic transduction could indeed contribute to our findings (Zhang et al., 2023). Collectively, these results imply that individuals with MS exhibit autonomic dysfunction affecting appropriate adjustments to peripheral resistance in inactive tissue during a mild sympathoexcitatory stimulus, which could contribute to a reduced ability to perform physical work.

Physiological changes associated with aging, such as increases in SBP, MAP, and PP can impair the ability to respond effectively to abrupt hemodynamic shifts. These changes are likely attributable to alterations in arterial structure and myocardial tissue, which affect vascular compliance and cardiac contractility. Furthermore, clinical studies of age‐related biomarkers suggest that biological aging is accelerated in individuals with MS compared to age‐ and sex‐matched counterparts without MS (Barcelos et al., 2019). Interestingly, despite the significant age difference between groups in this sample, age did not serve as a significant covariate. However, this finding warrants further investigation in this population, given the known deviations in aging patterns observed in individuals with MS compared to the general population.

Multiple sclerosis is characterized by a neuroinflammatory response, leading to a state of chronic inflammation and subsequent neurodegeneration (Choi et al., 2018). Multiple human studies have shown evidence of mitochondrial dysfunction in patients with MS, revealing that these disruptions in mitochondrial function are associated with significant changes in the morphology of neuroinflammation (Soilu‐Hänninen et al., 2005; Witte et al., 2013). This suggests that mitochondrial dysfunction may create an energy imbalance, thereby altering BF regulation. Research has also demonstrated elevated levels of C‐reactive protein, a marker of the innate inflammatory response, during relapses and disease progression in individuals with MS (Casas et al., 2008). Given that both C‐reactive protein and interleukin 6 (IL‐6) are common inflammatory markers associated with increased morbidity and mortality in the general population (via mitochondrial dysfunction), one would expect these markers to be elevated in individuals with relapsing–remitting MS (Baynard et al., 2018). However, this does not seem to be the case, as neither C‐reactive protein, nor IL‐6 differed between individuals with and without MS at rest in two separate studies conducted within our lab (Dinenno et al., 2001; Ranadive et al., 2012). Interestingly, these results were observed despite an increase in conduit and resistance arterial stiffness in individuals with MS compared to those without MS (Ranadive et al., 2012). Further research is needed to clarify the role of mitochondrial dysfunction in altered BF regulation and its impact on physical work capacity in individuals with MS.

### 4.1 Study limitations

This observational study involved a relatively small cohort, which limits the generalizability of the findings to the broader population of individuals with MS and predisposes our findings to the possibility of Type II errors. Consequently, these results are most applicable to individuals with relapsing–remitting MS and similar levels of disability. Recruiting a large sample of individuals with MS who had mild or moderate disability based on EDSS during a vulnerable health pandemic (i.e., COVID‐19) posed significant challenges. As a result, there is a notable age difference between participants with and without MS in this sample. However, the influence of age on our findings is likely minimal (and did not affect our outcomes) as older age is typically associated with reduced exercising limb blood flow‐ an effect not observed in our data (Carter et al., 2009). Moreover, although overall blood flow was higher in the MS group, the exercise‐induced response was similar between groups, and this difference disappeared when controlling for age. In addition, while the study was not powered for subgroup analyses, we conducted a sensitivity analysis using an age‐matched subset (*n =* 13 without MS; *n =* 10 with MS), which confirmed that age did not significantly affect the results. These findings, presented in Table S2, align with the analysis of covariance indicating that age was not a contributing factor. Nonetheless, this remains an important consideration for future studies designed to examine age‐related effects more directly.

This study used a small‐muscle‐mass handgrip model designed to engage the sympathetic nervous system in a population susceptible to fatigue. Consequently, these findings may not generalize to whole‐body exercise, such as lower‐limb protocols. Future studies should investigate this phenomenon using larger muscle groups and across a broader range of disability. We did not randomize the order of handgrip exercise intensities; however, the linear increase in blood volume observed here suggests that fatigue did not confound the active‐limb results. Finally, we did not control for menstrual cycle phase in our female participants due to mixed evidence regarding the influence of hormonal fluctuations on physiological responses to LBNP (Shankhwar et al., 2024). Additionally, recruitment challenges necessitated prioritizing participant inclusion to ensure adequate representation rather than excluding individuals based on cycle phase; however, information on contraceptive use is included in Table S1.

## 5 CONCLUSIONS

The current results provide experimental evidence by which individuals with MS are unable to appropriately control skeletal muscle blood flow in the inactive limb during exercise in the presence of mild sympathoexcitatory stimuli, demonstrated by a slight increase in blood flow to their inactive limb during handgrip exercise with the −20 mmHg LBNP stimulus. Furthermore, blood flow to the working limb of individuals with MS was similar to individuals without MS, suggesting adequate muscle perfusion during submaximal unilateral dynamic handgrip exercise, despite a reduced change in driving pressure during exercise in conjunction with the −20 mmHg LBNP sympathoexcitatory stimulus.

## AUTHOR CONTRIBUTIONS

Conceptualization and experimental design: SRS, NSL, TB, BF, and BAH. Data curation and experimental implementation: SRS, NSL, and BAH. Formal analysis, data visualization, and original draft preparation: SRS. Project administration, resource provision, and supervision: RWM, ATR, TB, and BF. Funding acquisition: TB and BF. All authors contributed to the manuscript's text and content, including revisions and edits. All authors have reviewed and approved the final version and agree to be accountable for all aspects of the work, ensuring the accuracy and integrity of the data through their critical input and intellectual oversight.

## FUNDING INFORMATION

Department of Defense Grant (W81XWH1810466) to BF.

## CONFLICT OF INTEREST STATEMENT

The authors report no conflicts of interest.

## ETHICS STATEMENT

Not applicable.

## Supporting information


Figure S1.

Figure S2.

Table S1.

Table S2.

## Data Availability

Data beyond what is presented in this manuscript is not publicly available but may be provided upon reasonable request to the corresponding author.
