# Supplementary material for: Impaired inactive limb blood flow regulation in adults with multiple sclerosis during sympathoexcitatory stimuli
Source: Physiol Rep. 2025 Dec 7;13(23):e70694. doi: 10.14814/phy2.70694 (PMC12682930; doi:10.14814/phy2.70694)
Supplement: Supplementary file 1 — Figure S1. [file PHY2-13-e70694-s003.docx]

Supplemental Materials


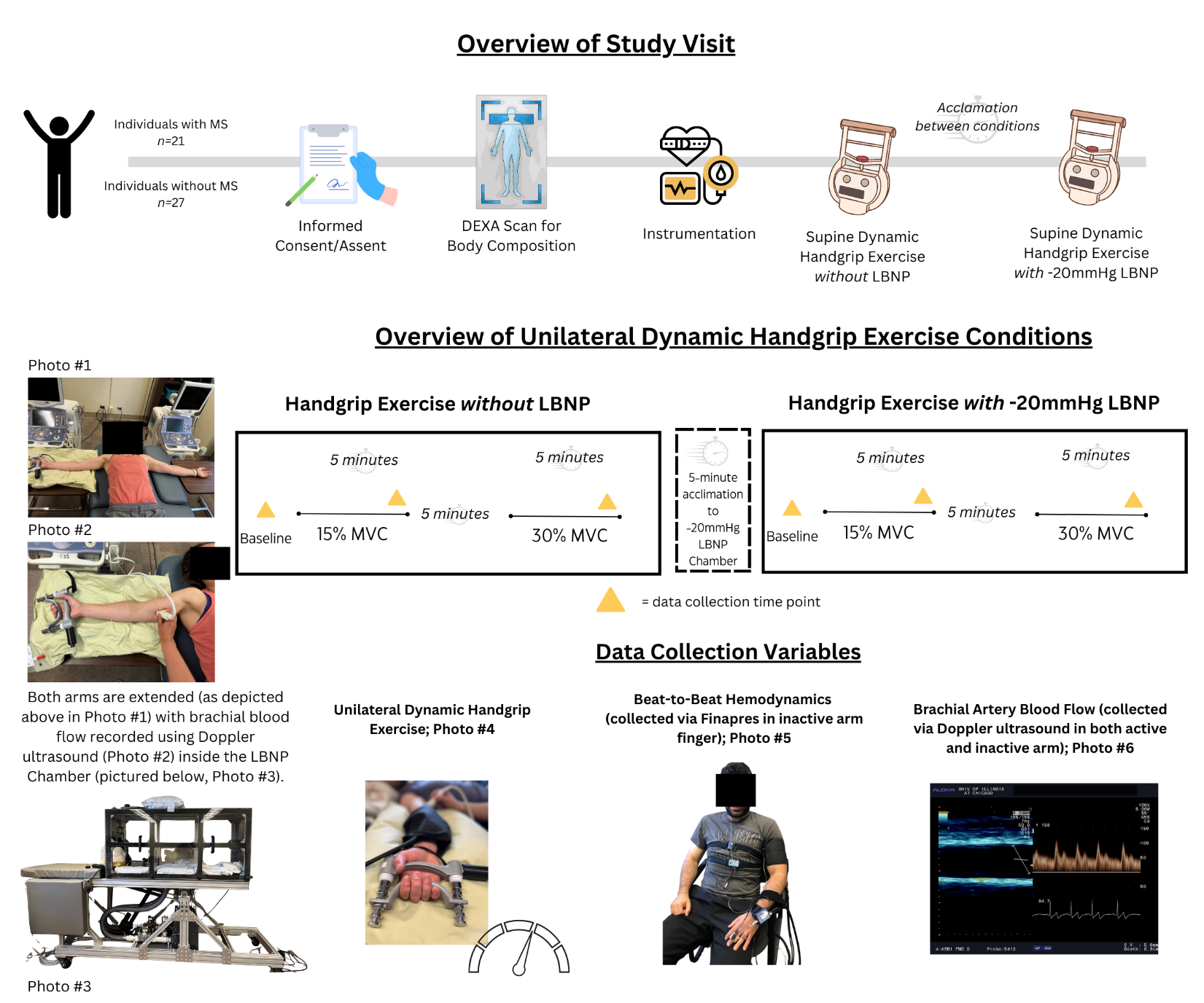


**Figure S1.** Experimental protocol employed for the current study.

Schematic and equipment used in the current study protocol investigating forearm blood flow in individuals with and without multiple sclerosis (MS). Unilateral dynamic handgrip exercise was performed with and without -20 mmHg lower body negative pressure (LBNP) to elicit a mild sympathoexcitatory stimulus. Each participant completed two handgrip trials at 15% and 30% of maximal voluntary contraction (MVC), under both LBNP and non-LBNP conditions. Handgrip cadence was standardized at 15 contractions per minute (2-second contraction, 2-second relaxation) and a custom gauge was projected on the ceiling to provide visual feedback. Photo #1 shows the supine position used for all trials. Photo #2 illustrates the active arm supported on a vac-lok pillow during handgrip exercise, with Doppler ultrasound performed on the brachial artery. Photo #3 displays the LBNP chamber used for testing. Additional photos depict the equipment used for data collection, including the handgrip device with an example of the gauge that was projected on the ceiling (Photo #4), beat-to-beat hemodynamic monitoring (Photo #5), and Doppler ultrasound system images (Photo #6). Images are representative of the experimental setup using models and do not include actual study participants or data. All photos were taken by SRS in the Integrative Physiology Laboratory at the University of Illinois Chicago and are used with permission.
