# Supplementary material for: Impaired inactive limb blood flow regulation in adults with multiple sclerosis during sympathoexcitatory stimuli
Source: Physiol Rep. 2025 Dec 7;13(23):e70694. doi: 10.14814/phy2.70694 (PMC12682930; doi:10.14814/phy2.70694)
Supplement: Supplementary file 2 — Figure S2. [file PHY2-13-e70694-s002.docx]

**
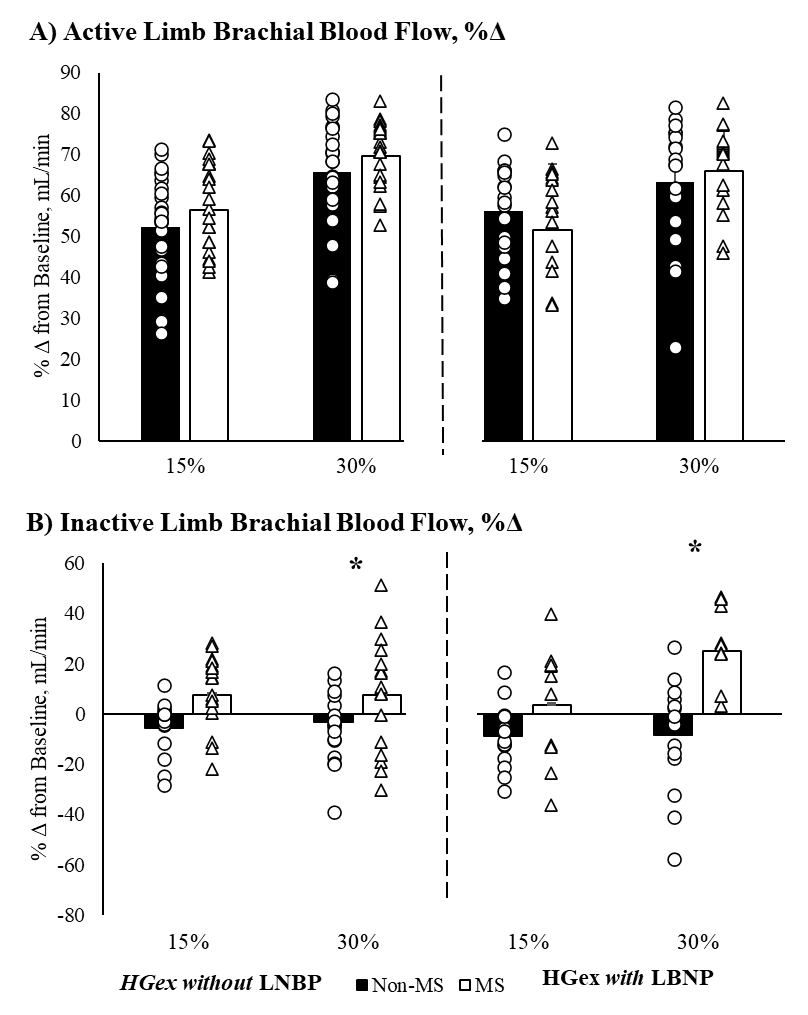
Supplemental** **Materials**

**Figure S2.** Percent change in brachial blood flow for the active (A) and inactive (B) limbs across all time points. As in Figures 1–3, dark bars represent mean percentage change for participants without MS, and open bars represent those with MS. Percentages below the bars indicate handgrip exercise intensities corresponding to (%Δ) Baseline to 15% and (%Δ) Baseline to 30% of maximal voluntary contraction, shown with (left of dotted line) and without (right of dotted line) the −20 mmHg LBNP stimulus.

*p<0.001 in a paired-samples t-test at condition; HGex, dynamic handgrip exercise; LBNP, lower body negative pressure; MS, multiple sclerosis; ‘15%’ and ‘30%’ represent relative intensities of maximal voluntary contraction; Data presented mean ± standard deviation.
