# Supplementary material for: Impaired inactive limb blood flow regulation in adults with multiple sclerosis during sympathoexcitatory stimuli
Source: Physiol Rep. 2025 Dec 7;13(23):e70694. doi: 10.14814/phy2.70694 (PMC12682930; doi:10.14814/phy2.70694)
Supplement: Supplementary file 3 — Table S1. [file PHY2-13-e70694-s004.docx]

| **Table S1.** Disease-modifying therapies and contraceptive use reported by study participants. | | |
| --- | --- | --- |
| **Disease-Modifying Therapies Reported by Participants with MS** | | |
| Medication | *n* | % |
| Ocrelizumab | 6 | 28.5 |
| Natalizumab | 3 | 14.3 |
| Ofatumumab | 3 | 14.3 |
| Fingolimod^a^ | 2 | 9.5 |
| Diroximel Fumarate | 1 | 4.8 |
| Glatiramer Acetate | 1 | 4.8 |
| None | 5 | 23.9 |
| **Contraceptive Use Reported by all Female Participants** | | |
| Contractive Method | Non-MS *n* (%) | MS *n* (%) |
| Oral Route | 1 (6) | 1 (6) |
| Contraceptive Injection | 0 (0) | 1 (6) |
| Hormonal Intrauterine Device | 1 (6) | 1 (6) |
| Transdermal Contraceptive Patch | 1 (6) | 0 (0) |
| None | 14 (82.4) | 14 (82.4) |
| MS, multiple sclerosis; ^a^, Fingolimod is associated with increased risk of cardiovascular events. | | |

**Supplemental Materials**
