# Supplementary material for: Impaired inactive limb blood flow regulation in adults with multiple sclerosis during sympathoexcitatory stimuli
Source: Physiol Rep. 2025 Dec 7;13(23):e70694. doi: 10.14814/phy2.70694 (PMC12682930; doi:10.14814/phy2.70694)
Supplement: Supplementary file 4 — Table S2. [file PHY2-13-e70694-s001.docx]

| **Table S2.** Descriptive characteristics and statistics of *a posteriori* subgroup analysis of age-matched participants. | | | | | | |
| --- | --- | --- | --- | --- | --- | --- |
| **A) Descriptive Characteristics for Subgroup Analysis of Age-Matched Participants** | | | | | | |
| **Variable** | **Non-MS** | | **MS** | | ***p*** | |
| *n* (M/F) | 13 (5/8) | | 10 (2/8) | | 0.34 | |
| Age (years) | 32 ± 3 | | 33 ± 3 | | 0.254 | |
| EDSS Score | - | | 3.2(2)ª | | - | |
| Race/Ethnicity | White: 12;  Black: 1 | | White: 8;  Latino: 1;  Asian: 1 | | 0.325 | |
| BMI (kg/m^2^) | 26.1 ± 4.1 | | 26.9 ± 6.5 | | 0.706 | |
| MVC (kg) | 83 ± 23 | | 75 ± 20 | | 0.401 | |
| **B) Active Limb Blood Flow** | | | | | | |
| Handgrip Exercise *without* -20mmHg LBNP | | | | | | |
|  |  |  |  | *p* | | |
|  | *n* | **Non-MS** | **MS** | **Time** | **Group** | **GxT** |
| Baseline | Non-MS: 10;  MS: 9 | 81.7 ± 13.2 | 75.9 ± 17.8 | <0.001 | 0.453 | 0.454 |
| 15% MVC |  | 164.8 ± 41.1 | 190.4 ± 82.1 |  |  |  |
| 30% MVC |  | 234.3 ± 71.4 | 265.6 ± 99.2 |  |  |  |
| Handgrip Exercise *with* -20mmHg LBNP | | | | | | |
|  |  |  |  | *p* | | |
|  | *n* | **Non-MS** | **MS** | **Time** | **Group** | **GxT** |
| Baseline *with* LBNP | Non-MS: 7;  MS: 7 | 84.0 ± 38.2 | 76.7 ± 12.4 | <0.001 | 0.852 | 0.598 |
| 15% MVC *with* LBNP |  | 171.1 ± 55.6 | 190.8 ± 57.5 |  |  |  |
| 30% MVC *with* LBNP |  | 294.1 ± 35.7 | 287.4 ± 84.9 |  |  |  |
| **C) Inactive Limb Blood Flow** | | | | | | |
| Handgrip Exercise *without* -20mmHg LBNP | | | | | | |
|  |  |  |  | *p* | | |
|  | *n* | **Non-MS** | **MS** | **Time** | **Group** | **GxT** |
| Baseline | Non-MS: 13;  MS: 10 | 65.4 ± 10.2 | 85.7 ± 17.8 | 0.072 | <0.001 | 0.080 |
| 15% MVC |  | 62.0 ± 8.4 | 99.0 ± 22.0 |  |  |  |
| 30% MVC |  | 66.8 ± 9.7 | 104.9 ± 27.1 |  |  |  |
| Handgrip Exercise *with* -20mmHg LBNP | | | | | | |
|  |  |  |  | *p* | | |
|  | *n* | **Non-MS** | **MS** | **Time** | **Group** | **GxT** |
| Baseline *with* LBNP | Non-MS: 10;  MS: 8 | 61.1 ± 10.3* | 84.9 ± 22.6 | 0.008 | <0.001 | 0.004 |
| 15% MVC *with* LBNP |  | 56.3 ± 14.3* | 98.4 ± 28.3 |  |  |  |
| 30% MVC *with* LBNP |  | 53.8 ± 9.0* | 114.6 ± 22.6 |  |  |  |
| Age-matching has been completed *a posteriori* for this subgroup analysis due to age differences in original sample; ªEDSS score is reported as mean (interquartile range);* Non-MS *lower* than MS at time point, *p<*0.001; BMI, body mass index; EDSS, Expanded Disability Status Scale; GxT, group x time interaction; LBNP, lower body negative pressure; M, male; MS, multiple sclerosis; MVC, maximal voluntary contraction; F, female; Data presented mean ± standard deviation, unless otherwise noted. | | | | | | |

**Supplemental Materials**
